# Zero-profile implant versus conventional cage-plate implant in anterior cervical discectomy and fusion for the treatment of degenerative cervical spondylosis: a meta-analysis

**DOI:** 10.1186/s13018-015-0290-9

**Published:** 2015-09-17

**Authors:** Haiyu Shao, Jinping Chen, Bin Ru, Feifei Yan, Jun Zhang, Shaonan Xu, Yazeng Huang

**Affiliations:** Department of Orthopaedics, Zhejiang Provincial People’s Hospital, No.158, Shangtang Road, Hangzhou, 310000 Zhejiang China; Department of Medicine, Zhejiang University, No.268, Kaixuan Road, Hangzhou, 310000 Zhejiang China

**Keywords:** Zero-profile, Anterior cervical discectomy and fusion, ACDF, Dysphagia, Degenerative cervical spondylosis

## Abstract

**Background:**

Zero-profile implant has become more and more popular in anterior cervical discectomy and fusion (ACDF) for the treatment of degenerative cervical spondylosis. However, there was no enough evidence judging its efficiency and safety. The aim of this analysis was to evaluate the efficacy and safety of Zero-profile implant compared with conventional cage-plate (CCP) in ACDF.

**Methods:**

All studies directly comparing the outcomes between the Zero-profile implant and CCP implant in ACDF were included, and the search strategy followed the requirements of the Cochrane Library Handbook. Two of the authors extracted relevant data and checked the accuracy independently using standardized data collection form.

**Results:**

Seven studies involving 560 patients were included, 262 in the Zero-profile group and 298 in the CCP group. Zero-profile implant had a lower rate of postoperative dysphagia at 2 weeks, 6 months, and 1 year (*p* = 0.0002, *p* = 0.008, and *p* = 0.001, respectively) than CCP implant. Zero-profile also reduced blood loss (*p* = 0.0001), while operation time and incidence of postoperative transient dysphagia had no statistical significance (*p* = 0.92, *p* = 0.42, respectively) between two groups.

**Conclusion:**

Based on the results of our analysis, the application of Zero-profile implant in ACDF had a lower rate of postoperative dysphagia at 2 weeks, 6 months, and 1 year than CCP implant. Zero-profile implant also had fewer blood loss during operation. More rigorous and adequately powered prospective randomized controlled trials with larger sample size are required to elucidate a more objective outcome.

## Introduction

Since anterior cervical discectomy and fusion (ACDF) was first described by Smith and Robinson [1], the procedure has become the gold-standard operation for single- or multiple-level degenerative cervical spondylosis. The application of stand-alone cage with a titanium plate (conventional cage-plate (CCP)) in ACDF has become more and more popular since studies [2–5] showed that CCP had many advantages compared with the stand-alone cage: improving sagittal alignment, interbody fusion rate, and stability and preventing interbody graft dislocation. However, complications related to the plates were not rare, such as postoperative dysphagia, adjacent level degeneration, and soft tissue injury [6–8]. And, sometimes, it seems to be inescapable.

Thus, Zero-profile implant was invented to reduce part of the complications. It consisted of a cage and a plant with locking screws which could be fixed into the intervertebral space to keep away from the front tissue [9, 10]. In the past few years, Zero-p implant has been used more and more wildly since studies [11–13] showed that it had many advantages over CCP implant.

The purpose of this analysis was to conduct a meta-analysis of relevant studies directly comparing the Zero-profile implant (Zero-profile group) with CCP implant (CCP group) in ACDF for degenerative cervical spondylosis.

## Methods

### Literature search

Electronic databases (Pubmed, EMBASE, Cochrane Central Register of Controlled Trial, and ISI Web of Science) were searched by two independent investigators (BR and FY). Results were last updated on October 2014. Boolean operators were used as follows: (zero-profile OR zero profile OR zero-p OR zero p) AND ((anterior AND fusion AND cervical AND (discectomy OR microdiscectomy OR Discectomies OR Diskectomies)) OR ACDF) with no restriction of publication year and language. We also hand-searched the reference lists of manuscripts included in order to detect other reports not identified by our original search.

### Inclusion and exclusion criteria

Inclusion criteria of degenerative cervical spondylosis were patients with degenerative disease and radiculopathy and/or myelopathy who had not responded to conservative treatment for at least 6 weeks. Exclusion criteria were developmental stenosis, ossification of the posterior longitudinal ligament, and a previous history of cervical spinal surgery.

Two of the authors independently reviewed the titles and abstracts and strictly followed the following inclusion criteria: (1) a direct comparison between the Zero-profile implant and CCP implant in ACDF with clinical and/or radiological outcomes, such as postoperative dysphagia scores, operation time, blood loss, Japanese Orthopaedic Association (JOA) score, visual analog scale (VAS) score, Cobb angle, and so on; (2) prospective, retrospective controlled trial; (3) the participants of the two groups without significant difference in age and gender; and (4) the language was English. Studies without a comparator, editorials, reviews, animal studies, and in vitro studies were excluded.

### Data extraction

Two of the authors extracted relevant data and checked the accuracy independently using standardized data collection form. The extracted data from each study included the following: first author, published year, study design, patient demographics (sample size, age, gender), follow-up time, and outcomes. If the data were not reported in the original article or not displayed in the table, we extrapolated them from the accompanying graphs. We also tried our best to contact the corresponding authors of the eligible trials to get any further useful data for our analysis. When the two reviewers had disagreements, one or more reviewers joined in discussion until consensus was achieved.

## Statistical analysis

We divided studies into two subgroups, retrospective study subgroup and prospective study group. All data were conducted with RevMan 5.0 analysis software (The Cochrane Collaboration, Copenhagen, Denmark). Odds Ratios (ORs) and 95 % confidence intervals (CIs) were used for the analysis of dichotomous outcomes. For continuous data, weighted mean differences (WMDs) were calculated with 95 % CI as the summary statistics. A chi-square test and *I*^2^ test were used to calculate the statistical heterogeneity. We considered *I*^2^ values of 25, 50, and 75 % as low, medium, and high heterogeneity, respectively. If *I*^2^ <50 %, we used the fixed-effects model; otherwise, the random-effects model was used. We performed such sensitivity analyses only if there were three or more studies included in the comparison.

## Results

### Literature search

**Fig. 1:**
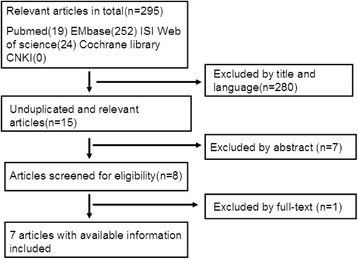
Flow diagram for the selection of studies

Literature search initially yielded 295 relevant citations. After titles and abstracts were reviewed, only eight articles met the criteria for inclusion in the report. And, one of them without eligible data of outcomes was excluded. Finally, seven studies [11–17] that met the predetermined eligibility criteria were included in this meta-analysis. The process of selecting studies was shown in Fig. 1.

### Characteristics of included studies

The characteristics of seven studies were presented in Table 1. There were two retrospective studies [13, 16], two prospective studies [15, 17], one prospective RCT [12], and two retrospective cohort studies [11, 14] with a total of 560 patients, 262 in the Zero-profile group and 298 in the CCP group. The number of surgical level ranged from 1 to 3. There were no statistically significant differences for patient age, gender, and number of surgical level of all seven studies. The clinical outcomes mainly included operation time, blood loss, pre- and postoperative JOA scores, and postoperative dysphagia. And, the radiological outcomes mainly included pre- and postoperative Cobb angles, VAS scores, adjacent disc degeneration, and neck disability index (NDI). The mean follow-up time was more than 6 months.

**Table 1:** Characteristics of the studies included in this meta-analysis

Study	Design	Sample size (male/female)		Surgical level	Mean age (years)		Outcome	Follow-up time (m)		Zero-profile implant
		Zero-p	CCP		Zero-p	CCP		Zero-p	CCP	
Wang ZW et al. (2015) [11]	Retrospective cohort	30 (18/12)	33 (14/19)	1 to 2	56.8	54	Operation time, blood loss, NDI and JOA scores, Cobb angles, fusion rate, postoperative dysphagia, adjacent disc degeneration and instability, cervical lordosis	Mean 24.1, minimum 12	Mean 23.8, minimum 12	ROI-C, LDR, Troyes, France
Son DK et al. (2014) [13]	Retrospective	21	27	1	55.4	50.2	Operation time, blood loss, mJOA and achieved mJOA scores, mJOA recovery rate, prevertebral soft tissue thickness, postoperative dysphagia, adjacent disc degeneration	6	6	Zero-p; Synthes GmbH, Oberdorf, Switzerland
Wang ZD et al. (2014) [16]	Retrospective	22 (11/11)	25 (10/15)	1	50.9	53.7	Operation time, blood loss, JOA scores, JOA recovery rate, postoperative dysphagia, adjacent segment degeneration	Mean 33.6	Mean 33.2	Zero-p; Synthes GmbH, Oberdorf, Switzerland
Li YB et al. (2013) [12]	Prospective, RCT	23 (9/14)	23 (13/10)	1	49.3	50.1	Operation time, blood loss, exposure time to X-rays, JOA and VAS scores, postoperative dysphagia, fusion time, adjacent disc degeneration	24	24	Zero-p; Synthes GmbH, Oberdorf, Switzerland
Qi M et al. (2013) [14]	Retrospective cohort	83 (47/36)	107 (58/49)	1 to 3	43.6	44.9	Operation time, blood loss, NDI and VAS scores, fusion rate, graft migration or nonunion, Cobb angles, modified Swallowing Quality of Life scores	Mean 18.6	Mean 19.3	Zero-p; Synthes GmbH, Oberdorf, Switzerland
Vanek P et al. (2013) [15]	Prospective	44 (26/18)	33 (19/14)	1 to 2	50.2	51.8	NDI scores, Cobb C and Cobb S angles, 2-year radiological stability, dysphagia	Minimum 24	Minimum 24	Zero-p; Synthes GmbH, Oberdorf, Switzerland
Miao JH et al. (2013) [17]	Prospective	39 (23/16)	50 (29/21)	1 to 3	50.3	52.6	JOA and VAS scores, postoperative dysphagia, Cobb angles, 1-year operation effect, implant displacement, and vertebral instability	Mean 16.9		Zero-p; Synthes GmbH, Oberdorf, Switzerland

*NDI* neck disability index, *JOA* Japanese Orthopaedic Association, *VAS* visual analog scale

### Meta-analysis results

#### Operation time

Five studies [11–14, 16] reported operation time of one level. One of the five studies [12] divided patients of both the Zero-profile group and CCP group into radiculopathy and myelopathy subgroups. We combined the data from the two subgroups using the formula below:

$$ M\mathrm{combined}=\frac{N_1{M}_1+{N}_2{M}_2}{N_1+{N}_2} $$

$$ \mathrm{SDcombined}=\sqrt{\frac{\left({N}_1-1\right){{\mathrm{SD}}_1}^2+\left({N}_2-1\right){{\mathrm{SD}}_2}^2+\frac{N_1{N}_2}{N_1+{N}_2}\left({M_1}^2+{M_2}^2-2{M}_1{M}_2\right)}{N_1+{N}_2-1}} $$

**Fig. 2:**
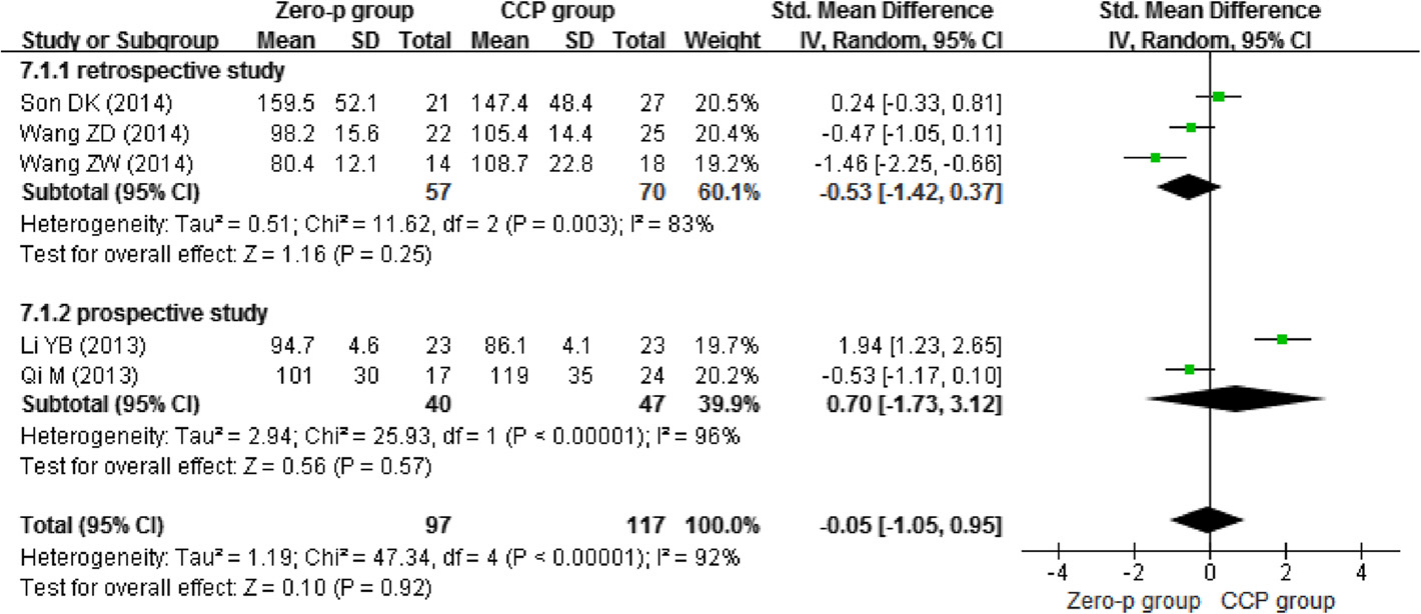
Forest plot of operation time of one level between the Zero-p group and CCP group

Three studies [11, 14, 16] showed that the Zero-profile implant could reduce operation time compared with CCP implant. However, the other two [12, 13] demonstrated contrary results. All data were pooled to make a meta-analysis. Due to the high heterogeneity (*I*^2^ = 92 %, *p* < 0.00001), we chose the random-effects model. We found that there was no significant difference in operation time (*n* = 214, *p* = 0.38) between the Zero-profile group and CCP group (Fig. 2).

### Blood loss

**Fig. 3:**
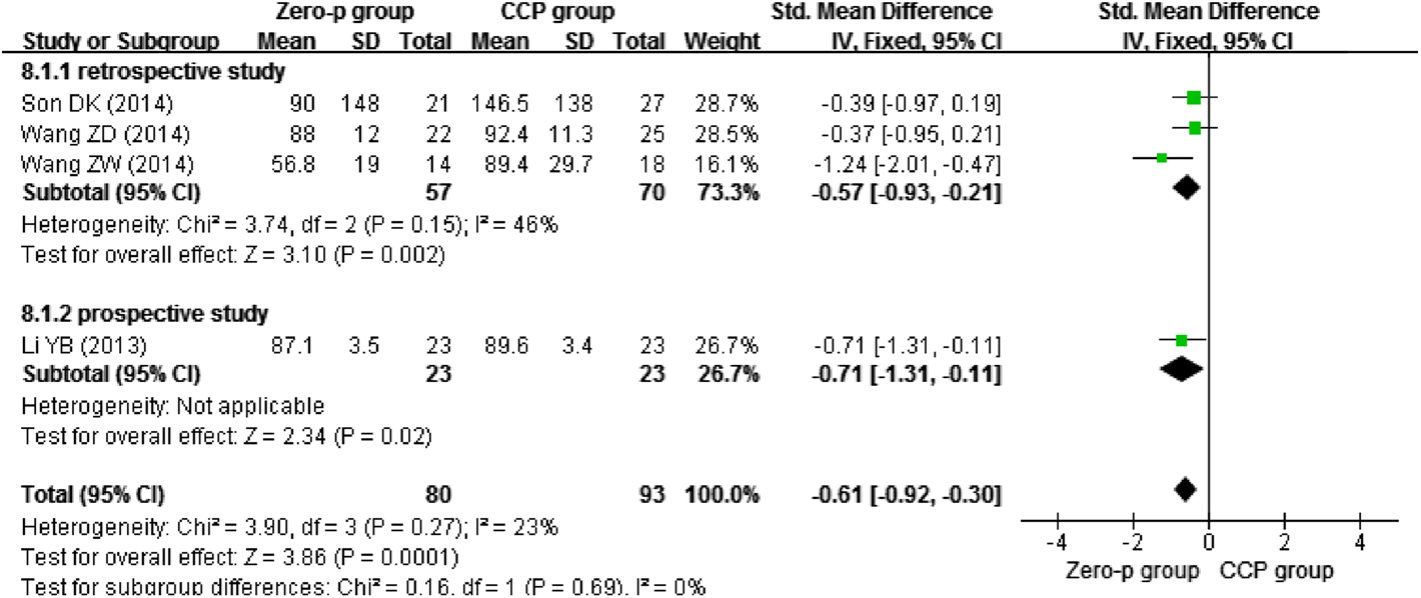
Forest plot of blood loss of one level between the Zero-p group and CCP group

Four studies [11–13, 16] reported the blood loss of one-level surgery. Again, one of them [12] divided patients of both the Zero-profile group and CCP group into radiculopathy and myelopathy subgroups. We combined the data from the two subgroups using the formula described above. We chose the random-effects model to combine the result due to the heterogeneity (*I*^2^ = 23 %). Figure 3 presents that Zero-profile implant had a statistical significance (*n* = 173, *p* = 0.0001) reducing blood loss, when compared with CCP implant.

### Postoperative dysphagia

**Fig. 4:**
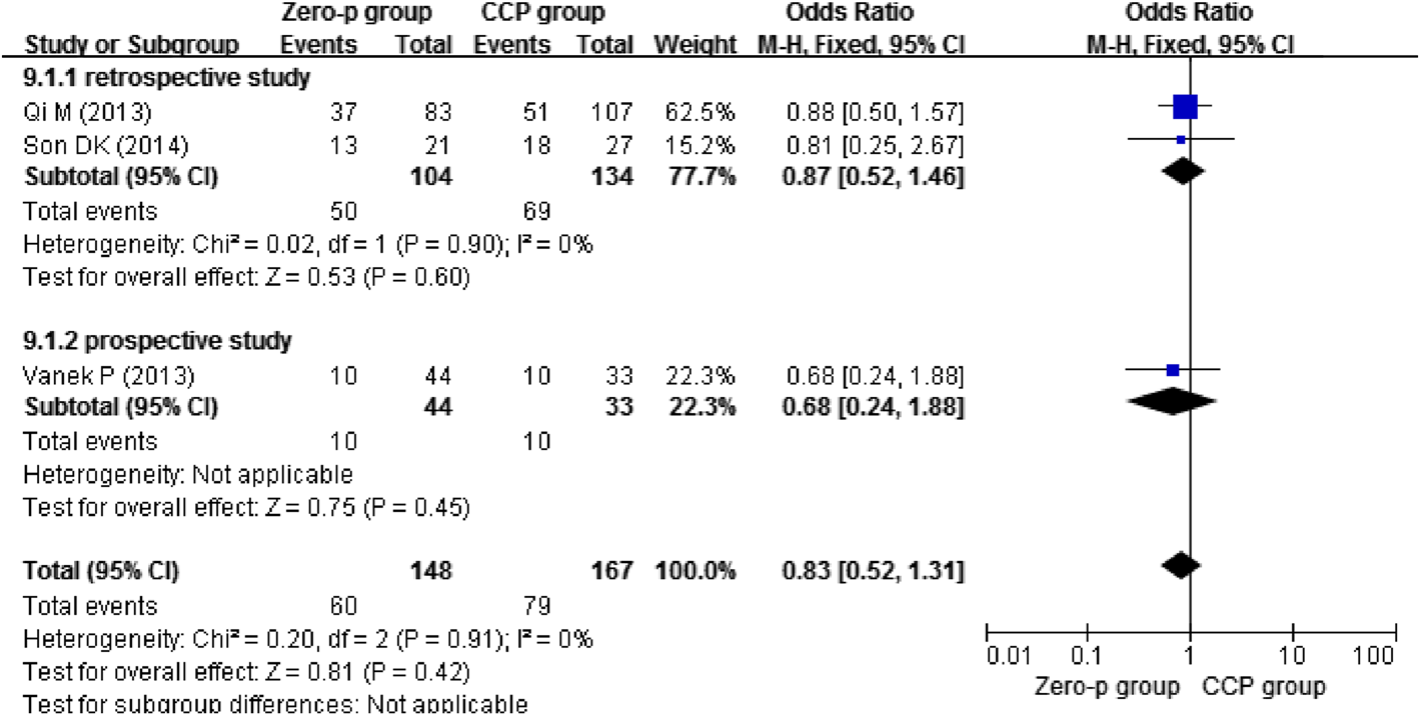
Forest plot of dysphagia at postoperative day between the Zero-p group and CCP group of all levels

**Fig. 5:**
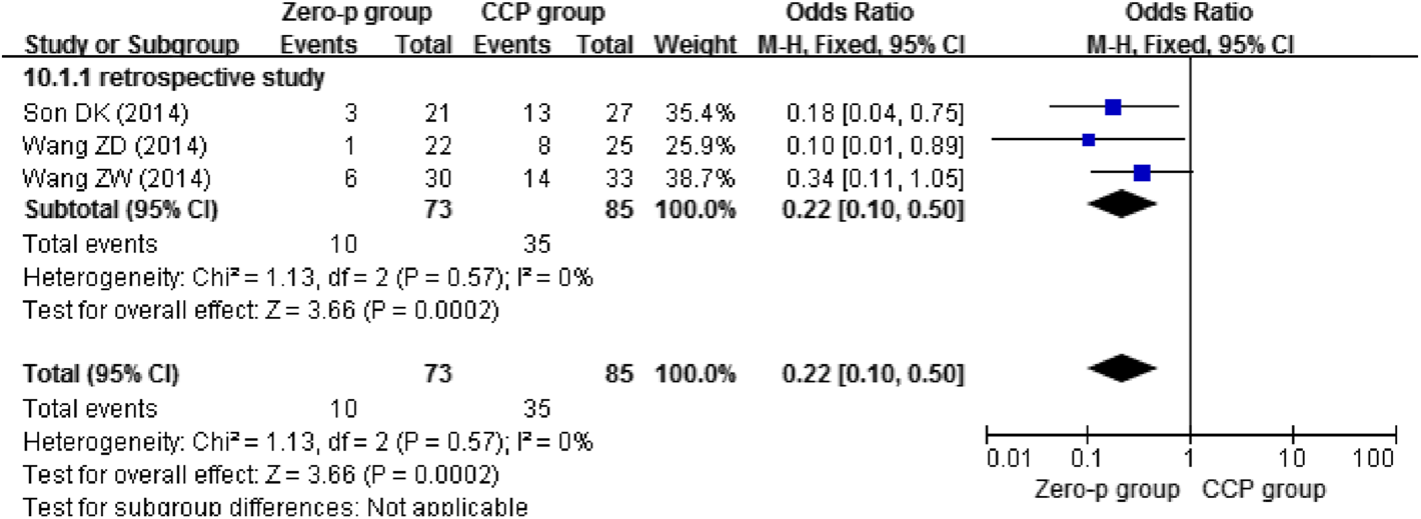
Forest plot of dysphagia at postoperative 2 weeks between the Zero-p group and CCP group of all levels

**Fig. 6:**
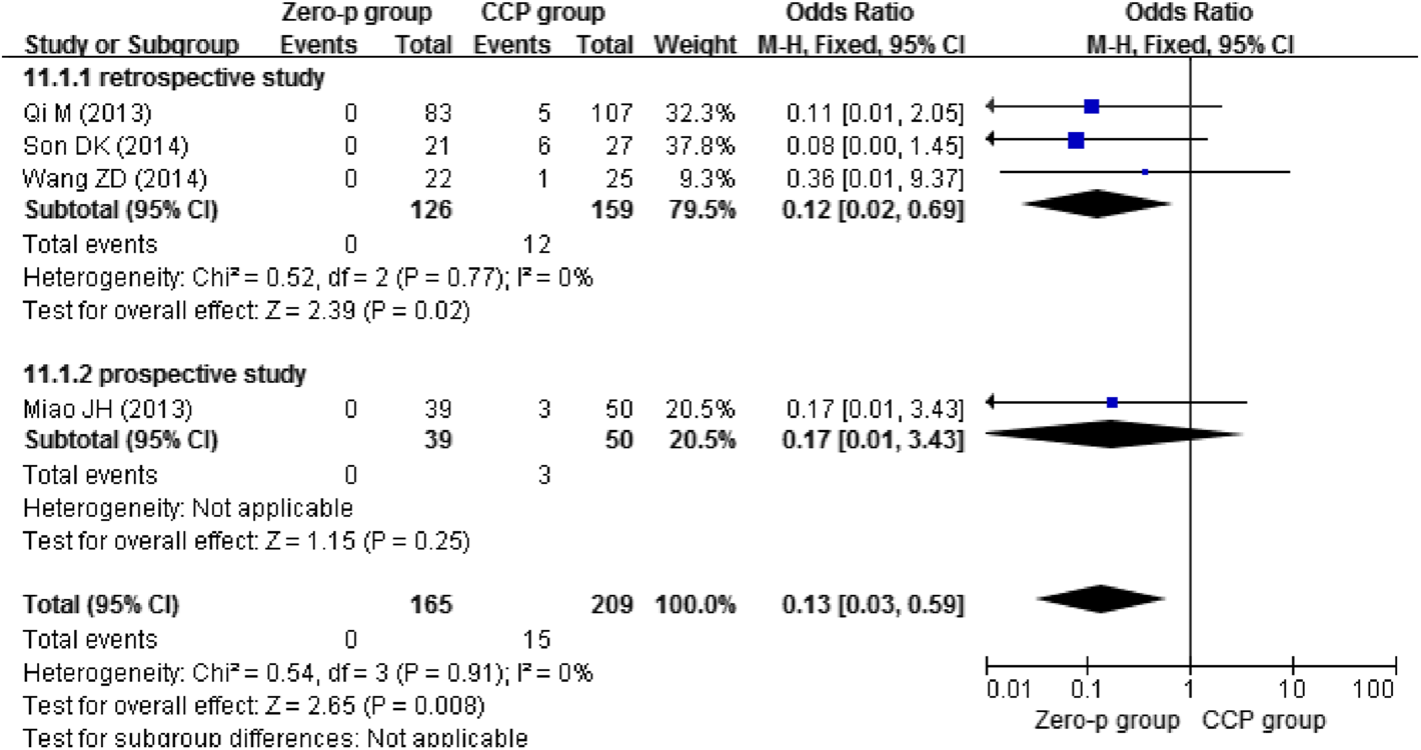
Forest plot of dysphagia at postoperative 6 months between the Zero-p group and CCP group of all levels

**Fig. 7:**
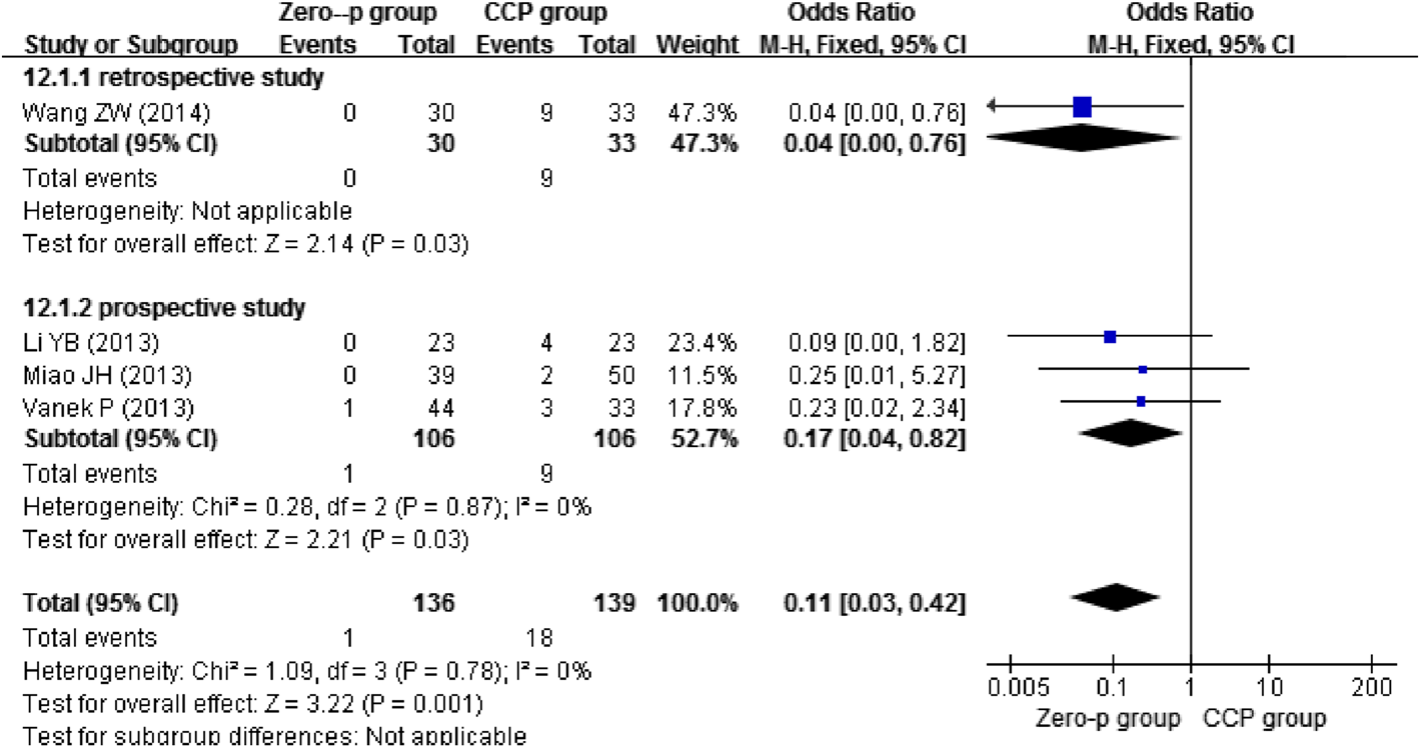
Forest plot of dysphagia at postoperative 1 year between the Zero-p group and CCP group of all levels

Three studies [13–15] reported postoperative day dysphagia, three studies [11, 13, 16] reported dysphagia at 2 weeks postoperative, four studies [13, 14, 16, 17] reported dysphagia at 6 months postoperative, and four studies [11, 12, 15, 17] reported dysphagia at 1 year postoperative. Due to the low heterogeneity (*I*^2^ = 0 %), we chose the fixed-effects model to combine the results. We found that the Zero-profile group had lower risk of postoperative dysphagia at 2 weeks (*n* = 158, *p* = 0.0002), 6 months (*n* = 275, *p* = 0.008), and 1 year (*n* = 374, *p* = 0.001), except postoperative day (*n* = 315, *p* = 0.42) as shown in Figs. 4, 5, 6, and 7.

### Other outcomes

There were less than three studies that reported the same outcomes or there were three or more studies that reported the same outcomes, but they were not of the same time, which made the results incomparable. So, we were unable to compare the operation time of two levels and three levels, JOA recovery rate, JOA, NDI, VAS scores, postoperative dysphagia of 2 and 3 months, fusion rate, Cobb angles, and other outcomes of Zero-profile implant and CCP implant in this analysis.

## Discussion

In the past several decades, there were many studies which suggested that CCP implant had many advantages compared with stand-alone cage. But, the application of anterior cervical plate may face with some complications. Esophageal injury, soft tissue compression, and adhesion caused by the anterior plant could lead to neck pain, hoarseness, and dysphagia [18–20]. Bazaz et al. [21] found a high dysphagia incidence of 50.2, 32.2, 17.8, and 12.5 % at 1, 2, 6, and 12 months postoperative, respectively. Lee et al. [22] found that a thinner and smoother plant could reduce dysphagia incidence postoperative significantly by means of less touch to the soft tissue precervical.

To lessen potential complications while maintaining the benefits of anterior cervical plate, the Zero-profile implant was invented. Zero-profile implant is a kind of device that contains a stand-alone cage and several angle-controlled screws, which can be implanted into the intervertebral space. The screws could get into the vertebral body through the endplate, and they provide adequate stability and avoid the implant contact to the soft tissue precervical. These unique structures offer a fixation mechanism that is similar to the function of a plate and screws. Since Zero-p implant of Synthes GmbH Switzerland was approved by the US Food and Drug Administration in 2008, many studies have engaged in comparing the outcomes between the Zero-profile implant and CCP implant in order to pursue the optimal implant in ACDF for degenerative cervical spondylosis patients. However, the inconclusive debate still remains.

In this analysis, we focused on objectively comparing the operation time, blood loss, and postoperative dysphagia between the Zero-profile implant (both contain the Zero-p of Synthes GmbH Switzerland and ROI-C of France) and CCP implant in ACDF.

The design of Zero-profile implant avoids the need for any additional internal fixation implants and theoretically circumvents the aforementioned complications associated with anterior plate while providing the segmental rigidity necessary for cervical spine fusion. In recent years, some studies have evaluated the safety and efficacy of Zero-profile implant. Njoku et al. [23] reported that Zero-profile implant had comparable pain outcomes, functional outcomes, and radiographic fusion rates with CCP implant. Barbagallo et al. [24] found that Zero-profile implant had a significant improvement on outcomes such as NDI scores, postoperative dysphagia, and fusion time. And, Scholz et al. [10] showed that all patients had a reduction on VAS pain, and the incidence of chronic dysphagia was lower than the current literature, which indicated good outcomes of Zero-profile implant. Meanwhile, some studies [11–17, 25] directly compared the clinical and radical outcomes of Zero-profile implant with CCP implant in ACDF, which could provide better evidence for comparing Zero-profile implant with CCP implant.

Thus, in this meta-analysis, we summarized the current evidences about the operation time, blood loss, and postoperative dysphagia comparing Zero-profile implant with CCP implant. After integrating the results, we found that Zero-profile implant had advantages in reducing the incidence of postoperative dysphagia at 2 weeks, 6 months, and 1 year (*p* = 0.0002, *p* = 0.008, and *p* = 0.001, respectively) compared with CCP implant.

Although the exact pathophysiologic mechanism of dysphagia remains unknown, according to Fountas et al. [8], esophageal injury, postoperative soft tissue edema, adhesive formations around implanted cervical plates, and postoperative hematoma may be the possible explanations for dysphagia-related symptoms. The Zero-profile implant can be completely contained in the decompressed intervertebral space, not placed across the anterior vertebral body, avoiding the stimulus to the esophagus and other prevertebral soft tissues, preserving as many normal anatomical tissues as possible. Although it might not be clinically relevant, we still can find significant difference in blood loss (*p* = 0.0001) between the Zero-profile implant and CCP implant. This is possibly because fewer steps are needed to insert the Zero-profile implant, which has a one-step locking mechanism with simple insertion of the cage and tightness of the screws. The Zero-p device has a smaller volume than the CCP implant, which allows a smaller incision. The smaller incision allows more limited resection and less exposure and avoids mechanical stimulus to the related structures [13].

To the best of our knowledge, our analysis is the first meta-analysis to evaluate the efficiency and safety of Zero-profile implant compared with CCP implant in ACDF. It combines the results of existing studies and makes the results more practical. In this way, it may reduce the effect of publication bias. Moreover, the results are encouraging. Zero-profile implant may be a viable alternative in ACDF for degenerative cervical spondylosis patients.

We acknowledge limitations such as the following: (1) the inclusion of both retrospective and prospective studies, random and nonrandom studies; (2) only seven studies were included, and their sample sizes were relatively small; and (3) not all main outcomes were analyzed in the analysis, such as JOA scores, Cobb angles, NDI scores, and fusion rate. These all could lead to bias and reduce the level of evidence in our analysis. (4) As listed in Fig. 1, our study contained two kinds of Zero-profile devices. Although they were of similar mechanism and structure, it may affect the results.

## Conclusion

Based on the results of our analysis, Zero-profile implant can reduce the incidence of postoperative dysphagia at 2 weeks, 6 months, and 1 year and reduce blood loss compared with CCP implant. It cannot reduce the operation time. In summary, the application of Zero-profile implant is superior to CCP implant in ACDF. Given possible biases in our study, more rigorous and adequately powered prospective randomized controlled trials with long-term follow-ups are required to elucidate a more objective outcome.

## Abbreviations

ACDF: anterior cervical discectomy and fusion
CCP: conventional cage-plate
JOA: Japanese Orthopaedic Association
mJOA: modified JOA
NDI: neck disability index
VAS: visual analog scale
